# Symptomatic reactions, clinical outcomes and patient satisfaction associated with upper cervical chiropractic care: A prospective, multicenter, cohort study

**DOI:** 10.1186/1471-2474-12-219

**Published:** 2011-10-05

**Authors:** Kirk Eriksen, Roderic P Rochester, Eric L Hurwitz

**Affiliations:** 1 Chiropractic Health Institute, PC, Clinic Director, 2500 Flowers Chapel Road, Dothan, AL 36305, USA; 2 Chiropractic Spine Center of North Georgia, Inc., Clinic Director, 475 S. Washington, Street, Suite C, Clarkesville, GA 30523, USA; 3 John A. Burns School of Medicine, Department of Public Health Sciences, University of Hawaii, Mānoa, Honolulu, Hawaii 96822, USA

**Keywords:** Chiropractic, Adverse Effects, Symptomatic Reactions, Manipulation, Upper Cervical

## Abstract

**Background:**

Observational studies have previously shown that adverse events following manipulation to the neck and/or back are relatively common, although these reactions tend to be mild in intensity and self-limiting. However, no prospective study has examined the incidence of adverse reactions following spinal adjustments using upper cervical techniques, and the impact of this care on clinical outcomes.

**Methods:**

Consecutive new patients from the offices of 83 chiropractors were recruited for this practice-based study. Clinical outcome measures included 1) Neck pain disability index (100-point scale), 2) Oswestry back pain index (100-point scale), 3) 11-point numerical rating scale (NRS) for neck, headache, midback, and low back pain, 4) treatment satisfaction, and 5) Symptomatic Reactions (SR). Data were collected at baseline, and after approximately 2 weeks of care. A patient reaching sub-clinical status for pain and disability was defined as a follow-up score <3 NRS and <10%, respectively. A SR is defined as a new complaint not present at baseline or a worsening of the presenting complaint by >30% based on an 11-point numeric rating scale occurring <24 hours after any upper cervical procedure.

**Results:**

A total of 1,090 patients completed the study having 4,920 (4.5 per patient) office visits requiring 2,653 (2.4 per patient) upper cervical adjustments over 17 days. Three hundred thirty- eight (31.0%) patients had SRs meeting the accepted definition. Intense SR (NRS ≥8) occurred in 56 patients (5.1%). Outcome assessments were significantly improved for neck pain and disability, headache, mid-back pain, as well as lower back pain and disability (*p *<0.001) following care with a high level (mean = 9.1/10) of patient satisfaction. The 83 chiropractors administered >5 million career upper cervical adjustments without a reported incidence of serious adverse event.

**Conclusions:**

Upper cervical chiropractic care may have a fairly common occurrence of mild intensity SRs short in duration (<24 hours), and rarely severe in intensity; however, outcome assessments were significantly improved with less than 3 weeks of care with a high level of patient satisfaction. Although our findings need to be confirmed in subsequent randomized studies for definitive risk-benefit assessment, the preliminary data shows that the benefits of upper cervical chiropractic care may outweigh the potential risks.

## Background

Previous observational studies have shown that adverse events (AE) following spinal manipulative therapy (SMT) applied to the neck and/or back (i.e., local discomfort in treatment area, radiating symptoms, headache) are relatively common, although these reactions tend to be mild in intensity and self-limiting [1-6]. Clinical trials have also found minor AEs after patients undergoing a trial of SMT [7-12]. Some studies have looked at cervical SMT and the relationship of this care to AEs and clinical outcome measures. One investigation concluded that subjects with AEs were less satisfied with care, perceived less improvement in their neck symptoms, and had more pain and disability at all follow-up assessments [10]. Attempts have been made to find predictors for side effects from SMT [13]. Women are more likely to report AEs compared to men, and patients with long lasting problems are more likely to report reactions as well [3,13]. One study found that the use of cervical rotation with SMT, and a patient's work status were moderately associated with an increased incidence of AEs [14].

Adverse events have also been studied for pediatric patients receiving SMT and have found to be rare [15,16]. Miller and Benfield [16] studied 679 children under the age of three receiving SMT with 85% of parents reporting an improvement. Seven parents reported an AE with a reaction rate of approximately 1 child in 100, or one reaction reported for every 749 treatments. There were no serious complications resulting from chiropractic treatment in their study, although one systematic review of pediatric patients aged 18 or younger found 9 cases of serious AEs in the literature [15]. Adverse reactions have been found to occur more frequently following cervical SMT compared to cervical mobilization, yet various studies have failed to show a significant clinical benefit for either form of treatment [8,10,11,17]. Subjects reporting AEs have reported less satisfaction with care, and were less likely to experience clinically meaningful improvements in pain and disability [10].

Several investigations have studied the impact of cervical SMT on different clinical outcomes. While the literature is inconsistent, some studies have shown improvement in Numerical Rating Scales (NRS) and/or Neck Disability Index (NDI) scores that range from about 30-60% for SMT care over a period from 2 weeks to 12 months [5,8,10]. However, no prospective study has examined the incidence of AEs following spinal adjustments using exclusively upper cervical techniques (UCT), and the impact of this care on clinical outcomes.

UCTs have been used by chiropractors and taught in chiropractic colleges since the 1930s. The utilization of UCT has varied over the years, although in recent years less than 5% of the profession uses these methods in their practices according to some surveys [18]. However, it has been suggested that this select population of chiropractors is more likely than others not to respond to a survey mailed out by the National Board of Chiropractic Examiners [18]. All UCTs originate from Palmer upper cervical care and these techniques range from the Grostic Procedure (and its various offshoots i.e., National Upper Cervical Chiropractic Association, Orthospinology, Advanced Orthogonal, Atlas Orthogonal), to Blair (and other toggle techniques), as well as Knee Chest procedures (see Table 1). The alignment integrity of the upper cervical spine is specifically analyzed radiographically and with other methods. Patients are checked for postural distortion, palpatory tenderness, range of motion, and/or paraspinal thermometry that are used on a visit-by-visit basis [19,20]. Incidentally, this method of care has been represented in the peer-reviewed literature to a significant extent considering the relatively small number of practitioners using the various techniques [19-21]. Some authors have challenged some of the central tenets of this form of chiropractic care [22,23], although others have either rebutted or provided conflicting data [19,20,24-30].

**Table 1:** Doctor and patient frequencies, by type of UCT

Technique	Doctors	%	Patients	%	Technique Description
**Atlas/Advanced Orthogonal**	9	10.8	130	11.9	Atlas Orthogonality was founded by Roy Sweat, D.C. in 1981. Advanced Orthogonality was founded by Stan Pierce, D.C. in 2001. Both procedures use a side posture patient position with a solid mastoid support, segmental contact over and directed toward the C1 transverse process via a stationary stylus on a table mounted instrument. The force is on a specific pre-calculated vector generated by a percussion wave mechanism.

**Blair**	11	13.3	157	14.4	Blair technique was founded by Williams Blair, D.C. in 1960. This technique uses a side posture patient position on a drop headpiece toggle table, with the surface of the headpiece parallel to the floor. The doctor contacts the patient with his pisiform over the anterior, posterior, or inferior transverse process based upon the necessary correction. With the headpiece cocked, a toggle and 180° torque type correction is administered depending on pre-determined vertebral alignment variables.

**Knee Chest**	16	19.3	194	17.8	Knee Chest technique has been in use since B. J. Palmer, D.C. developed UC chiropractic in 1931. The patient is in a kneeling position with their head turned on a solid headpiece table. Segmental contact point is over the posterior arch and uses a toggle-torque-recoil type thrust.

**National Upper Cervical Chiropractic Association (NUCCA)**	24	26.5	303	27.8	NUCCA was founded by Ralph Gregory, D.C. in 1966. This procedure uses a side posture patient position with a solid mastoid or skull support. The segmental contact is over the C1 transverse process via the pisiform using a hand adjustment. The force is on a specific pre-calculated vector generated by a triceps pull.

**Orthospinology/Grostic Procedure**	15	18.1	231	21.2	The Grostic Procedure was developed by John F. Grostic, D.C. in the late 1930s. Orthospinology was founded by a group of doctors in 1977 and implemented instrument adjusting as well as manual adjusting. Both procedures use a side posture patient position with a solid mastoid support. The segmental contact is over and directed toward the C1 transverse process via a moving stylus on a table mounted or hand-held instrument or via the pisiform using a hand adjustment. The force is a single pulse on a specific pre-calculated vector generated by a solenoid or a manual cam accelerated mechanism for instruments or a triceps pull for hand adjustments.

**Spinal Orthopedic Neurological Advancement and Research (SONAR)**	3	3.6	30	2.7	SONAR was developed Thomas Elliott, Jr., D.C. who was a NUCCA practitioner. SONAR employs procedures for taking and analyzing x-rays. The SONAR instrument uses computer generated specific sound waves in a precise vector of the size, magnitude and torque required to reposition the upper cervical spine.

**Toggle Recoil/Duff**	5	6.0	45	4.1	Toggle Recoil was popularized in the 1930s by B.J. Palmer, D.C. with his development of HIO technique (which was also done in the Knee Chest position). This type of adjustment is made in the side posture patient position on a drop headpiece toggle table, with the doctor's pisiform contact over the C1 transverse process. A quick contraction and relaxation of the triceps generates the administered force. The Duff Method of Analysis was developed by Stephen A. Duff, Sr., D.C. utilizes a specific pre/post thermographic instrumentation procedure and upper cervical x-ray analysis. The adjustive technique utilizes a modified toggle-recoil to the atlas or axis with a predetermined vector and contact point. A side posture table with a drop mechanism is used.

**Total**	83	100	1090	100	

*9 doctors listed a secondary upper cervical procedure.

It must be noted that various terms have been used in previous studies to describe AEs following chiropractic treatment such as unpleasant reactions, side effects, or adverse reactions. However, this study will use the term 'symptomatic reactions' because some of the reported symptoms may not be considered 'adverse' in clinical practice (i.e, tiredness, lower back soreness) but could represent change or healing. It has been reported in one study that at least 1 positive 'side effect' results after SMT in 21 to 25% cases [31]. These nonmusculoskeletal responses included 'easier to breathe', 'improved digestive function', 'improved vision', and 'improved circulation'. Symptomatic reactions (SR) will be further operationally defined in the *Methods *section.

Little research is available in the published literature on the topic of side effects resulting from UCT. The authors of one case series and two randomized controlled studies using UCT reported no serious AEs [32-34]. The primary objective of this current report is to describe both SRs and clinical outcomes following a short term of chiropractic care that uses only UCT in a large cohort from multiple chiropractic offices.

## Methods

### Study Design and Source Population

A prospective, multicenter, practice-based cohort study was conducted for patients receiving only upper cervical (UC) chiropractic care. Subjects who fulfilled the inclusion criteria were recruited by chiropractors in their private practices and were followed for approximately two weeks of care. Each participating chiropractor was asked to recruit at least 10 consecutive new patients.

### Approval and Funding

The Institutional Review Board of Life University, Marietta, GA granted approval for the study. Funding was provided by allocated funds and donations from various individuals and chiropractic organizations to the Society of Chiropractic Orthospinology, Inc., a non-profit organization. All authors in this study were involved with the development of the study.

### Recruitment and Participation of Chiropractors

The first author and principal investigator (KE) contacted the presidents of all of the known UCT and related organizations. The basic protocol of the study was explained and cooperation was obtained by most all of the UCT groups. Announcements were made by most of the UCT leaders to their own memberships, as well as KE. This was done live at various conferences, but also via e-mail and print mail dissemination for recruitment of doctors in the study.

Participating chiropractors completed a questionnaire administered once at the beginning of the study relating to basic sociodemographic information concerning himself/herself and treatment practices (i.e., years of experience, how many weeks in practice annually, the approximate number of UC adjustments made each week, and the types of UCT used). Prior to the start of patient recruitment, all participating doctors had to either attend a live conference call or listen to a recording of the presentation. The first author (KE) provided a detailed review of the study protocol during the call, and was able to answer any questions as well. All doctors were provided with a written protocol of the study, and the participants were able to communicate with KE by phone or via e-mail. The doctors were encouraged to assign a Practice Manager (PM) to administer paperwork to the patients and help with other research activities. The doctor had to train the PM about the details of the study and monitor the PM's activities to make sure that the rigor of the study was upheld.

### Subject Recruitment and Inclusion/Exclusion Criteria

Patients were obtained through each practice's normal referral and marketing procedures. Subjects in this study were between 18 and 85 years of age. Pregnant subjects were not permitted to participate in this study due to the fact that patients had X-rays taken based on clinical indicators. During the 20-month period that chiropractors were invited to participate in the study and begin data collection, each treating doctor was asked to provide data from at least 10 consecutive new patients that agreed to participate in this study and met the inclusion criteria. Doctors assigned the first new patient that was approached and agreed to take part in the trial with the number '1'. Each successive new patient was assigned sequential numbers until 10 patients had completed the study. The doctor was required to provide a short explanation on a form to account for the exclusion of a sequenced new patient that declined to participate or failed to meet the inclusion criterion. Some chiropractors provided more than 10 cases, while some doctors had less than 10 patients that completed the study.

In order to further check for possible errors or data collection bias, a random 5% sample of the cases was examined by one of the co-investigators (RPR) of the study. The actual records provided by participating doctors as well as the data spreadsheets produced by KE were carefully reviewed by RPR in the location that they were stored (KE's office). Data entry was found to have a 99.87% accuracy. The audit report was reviewed by KE who corrected the data errors as well as checked other records.

### Data Collection and Clinical Outcome Variables

Participating patients were given an informed consent and HIPAA release form. The doctor explained the treatment protocol, which only included upper cervical care (i.e., no physical therapy or non-upper cervical adjusting/manipulation/mobilization for the duration of the study). If the patient agreed to participate they signed the informed consent and HIPPA release form, and a copy was provided for their own personal records. The HIPAA release form gave the doctor permission to release a copy of the patient's research data for statistical analysis. The following were assessed at baseline: age, sex, sociodemographics, the nature and severity of the presenting complaint(s), and the onset/duration of the patient's primary and secondary chief complaints.

To assess the impact of the patient's pain on their 'activities of daily living' the patient completed a Neck Disability Index (NDI) and/or Oswestry Disability Index (ODI), depending on whether the subject had symptoms in the neck or back, respectively. The NDI and ODI have been demonstrated to be reliable and valid instruments [35,36]. These are 10-question, multiple-choice surveys that are simple and quick to complete. The scores were calculated on a 100-point scale in this study. The patient rated their overall neck, headache, mid back and/or low back pain using a written 11-point Numeric Rating Scale (NRS), for applicable complaints (0 = no pain, 10 = the worst possible pain).

### Intervention

Patient management and visit frequency were left to the discretion of the chiropractor, although all doctors agreed to refrain from using any other type of spinal care (i.e., full spine manipulation, physical therapy, massage therapy) other than the upper cervical technique (UCT) that they were trained to use. Each doctor recorded the type of UCT they used on a standardized form. A list of UCT that were used in this study is provided in Table 1 and the number of participating doctors for each UCT.

### Re-examination Clinical Outcomes

The doctors were instructed to have the patient complete a follow-up NDI and/or ODI after approximately two weeks of care. The patients also provided a follow-up NRS for their neck, headache, mid back and/or low back pain (for all applicable symptoms) at the end of the treatment period.

### Patient Satisfaction

Satisfaction was measured at the end of the treatment period by a question that asked, "How satisfied are you with the treatment by your chiropractor?" An 11-point NRS (ranging from 0 = very dissatisfied to 10 = very satisfied) was privately scored by each patient. All of these forms were to be given to the patients by either the PM or they were filled out without the doctor present to help prevent any bias.

### Symptomatic Reactions

At the end of the treatment period, subjects were asked about possible SR. A survey was provided that asked if the patient had experienced any discomfort or unpleasant reaction that they felt was related to their chiropractic care, and not the continuation of a pre-existing symptom. However, the investigators did want to know if any of the patient's prior symptoms had worsened since undergoing care. The PM or doctor reviewed the survey to make sure the patient understood the questions and the purpose of the questionnaire. The patient then filled out the survey by rating SR or checking off if each symptom 'does not apply.' The following symptoms were provided on the questionnaire: 1) neck pain and/or stiffness/soreness, 2) radiating (arm or leg) pain/discomfort, 3) arm or leg weakness, 4) tiredness/fatigue, 5) headache, 6) dizziness/imbalance, 7) nausea/vomiting, 8) ringing in the ears, 9) blurred or impaired vision, 10) confusion or disorientation, 11) depression or anxiety, 12) fainting, 13) low back discomfort/soreness. A space was provided for the patient to fill in any other SR that was not provided in the survey.

The intensity of each SR was graded using an 11-point NRS. A question asked when the SR first started with the following choices: began less than 30 minutes, or 0.5 to 4 hours, or 4 to 24 hours, or more than 24 hours after receiving their adjustment. The duration of the SR had to be noted with the following choices: lasted less than 10 minutes, or 10 minutes to 1 hour, or 1 to 24 hours, or more than 24 hours. The patient was asked to indicate the impact on daily activities for each SR as well. Three choices were provided: affects life "not at all," or "a little," or "a lot."

The data analysis used a method similar to Rubinstein et al. [5] where each SR was defined as either 1) a new related complaint that was not present at baseline or 2) a worsening of the presenting complaint by >30% compared with baseline occurring <24 hours following care. The 30% cut-off point is based upon earlier work demonstrating that this represents the minimal clinically important difference on an 11-point NRS [37]. Intense SR were defined as any complaint fulfilling our definition of a SR that also scored ≥8 in intensity on the 11-point NRS. This term must not be confused with serious adverse event, which refers to events resulting in death, life-threatening situations, need for admittance to a hospital, or temporary or permanent disability.

After the patient completed the paperwork, the doctor reviewed the documents to make sure that all of the previous information was recorded accurately and completely. The chiropractor noted how many times the patient was seen once treatment began and how many times the upper cervical spine was adjusted during the treatment period. He/she also had to record all mediating clinical measurements after the patient's first upper cervical adjustment. The doctors answered a question in the survey concerning the occurrence of serious AE (i.e. the death or permanent impairment of a patient) resulting from any of their UC adjustments throughout their career. If so, they had to explain the incident. All patient and doctor data were entered into a Microsoft Excel spreadsheet.

### Data Analysis

Data were entered exported to SAS software (version 9.1, SAS Institute, Cary, North Carolina) for data cleaning, management and analysis. Frequencies, percentages and frequency distributions were computed for categorical variables; means, standard deviations, medians, and ranges were computed for continuous variables. Descriptive statistics were used to summarize patient demographic and health characteristics measured at baseline; symptomatic reaction and satisfaction data at follow-up; and characteristics of the participating chiropractors.

Absolute and percentage change scores from baseline to follow-up were computed for the NDI, ODI, and each NRS. Percentages of patients with subclinical outcomes (NDI/ODI scores of <10% and NRS <3) on these measures following treatment were also calculated for the total sample, as well as for the subgroups with baseline scores of at least 10% on the NDI and ODI and at least 3 on each NRS. SRs were categorized according to their 1) presence (yes or no), 2) start time and severity (≤within 24 hours and NRS >1), and 3) severity alone (>7). Worsening of the presenting or existing complaint by >30% based on the 11-point NRS for patients presenting with neck, headache, mid-back, or low-back pain was considered in sub-analyses.

We compared the frequencies of SR and levels of satisfaction according to duration of presenting complaint (acute [<3 weeks], subacute [3-13 weeks], and chronic [>13 weeks]). Risk ratios and 95% confidence intervals were used to estimate associations of episode duration with reported SR and satisfaction.

## Results

### Participating Upper Cervical Chiropractors

One hundred fifteen (115) UC chiropractors expressed interest in participating in this study by listening to the recorded directions and receiving a package of documents. Eighty-three (72.2%) doctors from 4 countries provided consecutive patients for this study. The United States was represented by 70 chiropractors from 29 states, and Canada had 11 doctors representing three provinces. Europe was represented by two chiropractors (one each from England and Spain). The mean years in practice was 13.0 (SD 10.5) with a median of 9 years. The UC doctors were asked to provide the average number of UC adjustments they administered per week as opposed to the number of office visits since patients may not require an adjustment on each office visit. The mean number of adjustments per week was 85.5 (SD 69.4) for these doctors. The number of weeks practiced per year was 48.7 (SD 3.6). The average number of career adjustments per doctor was 61,265 (SD 7077) with a total of 5,085,011 career adjustments for all participating UC chiropractors.

Statistics for the UCTs used during the study are listed in Table 1. Adjustments were administered by hand, hand-held or table mounted instruments. Two different biomechanical models are represented by these chiropractic UCT, i.e. the articular and orthogonal models. The orthogonal model was represented by 694 (63.7%) cases and 396 (36.3%) represented the articular model. Orthogonal UCTs included the following: Advanced Orthogonal, Atlas Orthogonal, Grostic Procedure, National Upper Cervical Chiropractic Association, Orthospinology, and Spinal Orthopedic Neurological Advancement & Research. Articular UCTs include the following: Blair, Duff, Knee Chest, and Toggle Recoil. Hand (or manual) adjustments were delivered in 732 (67.2%) cases, while 358 (32.8%) were adjusted by instrument.

### Study Population

One thousand ninety (1090) patients were recruited from June 5, 2007 through January 30, 2009. The mean age was 46.1 (SD 14.2) years. Three hundred ninety-one (391) were male (35.9%) and 699 were female (64.1%). A total of 4,920 office visits occurred with patients requiring 2,653 UC adjustments over an average of 17 days. The vast majority (73.5%) of cases were of chronic onset having mild to moderate disability and moderate pain. Patient descriptions, duration of presenting symptoms and care delivery variables are summarized in Table 2. Of all patients, 339 (31.1%) required only one upper cervical adjustment, 312 (28.6%) cases received 2 UC adjustments, 211 (19.4%) had 3 UC adjustments while 126 (11.6%) patients had 4 UC adjustments. The remaining 9.3% had more than 4 UC adjustments.

**Table 2:** Frequency distributions and/or means (SDs) of selected sociodemographic, clinical, and health-care variables

	N	Mean (SD)/Percent	
**Age**	1090	46.1 (14.2)	
**Male**	391	35.9%	
**Female**	699	64.1%	

**Duration**			
Acute <3 wks	139	12.85%	
Subacute 3-13 wks	148	13.68%	
Chronic >13 wks	795	73.48%	

**Care Delivery Variables**	**N**	**Mean (SD)**	**Median**
Follow-up (Days)	1085*	17.0 (6.7)	15.0
Office Visits	1090	4.5 (1.4)	4.0
UC Adjustments	1090	2.4 (1.4)	2.0

* Follow-up dates not available for 5 cases

Patients listed multiple symptoms or conditions fitting into 28 different chief complaints or reasons for seeking care from an UC chiropractor; however, 80.9% were due to spinal pain/dysfunction or headaches. Table 3 lists the frequencies of presenting conditions or reasons for patients seeking care.

**Table 3:** Frequency distributions of presenting chief complaints

Presenting Complaints	Primary	%	Secondary	%	Tertiary	%
**cervical pain/dysfunction**	382	35.24	173	21.25	28	9.59
**lumbo-pelvic pain**	299	27.58	176	21.62	66	22.60
**headaches**	141	13.01	110	13.51	42	14.38
**mid-back pain**	55	5.07	80	9.83	35	11.99
**lower extremity pain**	39	3.60	89	10.93	24	8.22
**shoulder pain**	27	2.49	52	6.39	35	11.99
**upper extremity**	23	2.12	48	5.90	19	6.51
**fibromyalgia**	20	1.85	15	1.84	3	1.03
**dysequilibrium**	19	1.75	14	1.72	7	2.40
**temporomandibular joint pain**	9	0.83	4	0.49	1	0.34
**facial pain/dysfunction**	7	0.65	2	0.25	0	0
**blood pressure**	7	0.65	0	0	2	0.68
**neurological disease**	7	0.65	6	0.74	2	0.68
**brain dysfunction**	6	0.55	3	0.37	2	0.68
**wellness care**	6	0.55	0	0	0	0
**other**	37	3.41	42	5.16	26	8.90
**Totals**	1084	100	814	100	292	100

Other: primary presenting complaints with < 0.50% occurrence: atopic disorders, diabetes, ear, GI dysfunction, hypothyroidism, Lyme's disease, postural distortion, psoriatic arthritis, psychological disorders, sinus problems, sleep disorders, thorax dysfunctions, visual disturbance. Other: secondary complaints are the same as the primary with the addition of: autoimmune disorder, irregular cycle and weak immune system.

### Clinical Outcomes

The clinical outcome measures are presented in Table 4. Neck pain improved over the 17-day treatment period that included an average of 2.4 upper cervical adjustments by a mean of 56.8% based on the changed NRS scores. This change is clinically and statistically significant (CSS). Headache pain improved by 62.8%, which is also a CSS difference. Thoracic and lumbar pain improved clinically and statistically significantly by 58.6% and 57.0%, respectively. Perceived neck disability as related to activities of daily living captured by the NDI improved by 13.3 points (100 point scale) (47.1%) at follow-up which is CSS. The ODI improved by 11.3 points (100 point scale) (45.0%) and is both a CSS difference.

**Table 4:** Frequency distributions and/or means (SDs) of clinical outcome variables at baseline and following an average of 17 days, 4.5 visits and 2.4 upper cervical adjustments

		Baseline			Follow-Up		Reached Sub-Clinical Status*	
Clinical Outcome								
	N		%	N		%		%
*Neck NRS*								
None (0-2)	127		15.01%	525		62.06%	Total Sample:	62.20%
Mild (3-4)	215		25.41%	199		23.52%	Pre ≥3	57.10%
Moderate (5-7)	338		39.95%	103		12.17%		
Severe (8-10)	166		19.62%	19		2.25%		
Mean (SD)		5.2 (2.4)			2.3 (2.1)	Δ3.0 (56.8%)		
								
HA NRS								
None (0-2)	136		20.54%	450		67.98%	Total Sample:	68.00%
Mild (3-4)	157		23.72%	118		17.82%	Pre ≥3	62.70%
Moderate (5-7)	208		31.42%	73		11.03%		
Severe (8-10)	161		24.32%	21		3.17%		
Mean (SD)		5.1 (2.7)			1.9 (2.3)	Δ3.2 (62.8%)		
								
*MBP NRS*								
None (0-2)	150		22.09%	460		67.75%	Total Sample:	67.90%
Mild (3-4)	209		30.78%	127		18.70%	Pre ≥3	63.00%
Moderate (5-7)	204		30.04%	76		11.19%		
Severe (8-10)	116		17.08%	16		2.36%		
Mean (SD)		4.7 (2.5)			2.0 (2.1)	Δ2.8 (58.6%)		
								
LBP NRS								
None (0-2)	124		14.90%	507		60.94%	Total Sample:	61.20%
Mild (3-4)	199		23.92%	163		19.59%	Pre ≥3	55.90%
Moderate (5-7)	283		34.01%	127		15.26%		
Severe (8-10)	226		27.16%	35		4.21%		
Mean (SD)		5.5 (2.6)			2.4 (2.3)	Δ3.2 (57.0%)		
								
NDI								
None (0-9)	75		10.40%	305		42.36%	Total Sample:	42.40%
Mild (10-29)	357		49.51%	316		43.89%	Pre ≥10%	37.60%
Moderate (30-49)	200		27.74%	77		10.69%		
Severe (≥50)	89		12.34%	22		3.06%		
Mean (SD)		28.2 (16.7)			14.9 (13.5)	Δ13.3 (47.1%)		
								
Oswestry								
None (0-9)	105		14.64%	315		43.93%	Total Sample:	43.90%
Mild (10-29)	361		50.35%	309		43.10%	Pre ≥10%	36.90%
Moderate (30-49)	174		24.27%	73		10.18%		
Severe (≥50)	77		10.74%	20		2.79%		
Mean (SD)		25.9 (17.1)			14.2 (13.8)	Δ11.6 (45.0%)		
								
Patient Satisfaction				1089	9.1(1.6)			

NRS = 0 to 10 numeric rating scale.

* Post subclinical scores for the NDI and OBI are <10%; post subclinical scores for the NRS measures are <3. (Net = Follow-up - Baseline)

The percentage of patients that returned to a sub-clinical status (SCS) at follow-up was also reported. Based on NRS for neck pain, 62.2% of the total sample was at a SCS after approximately 2 weeks of UC chiropractic care. Of those that had a baseline of ≥3 neck pain NRS (which is CSS pain), 57.1% achieved SCS. For headache patients the percentages were slightly higher with 68.0% at a SCS on follow-up and for those with CSS baseline pain (≥ 3 NRS) 62.7% reached SCS. Patients that presented with thoracic or lumbar pain reached SCS after a short trial of UC chiropractic care for 67.9% and 62.1%, respectively for the total sample. At follow-up, 42.2% reached SCS for cervical disability according to the NDI, and 37.6% of those patients with CSS (≥10 points) disability from neck pain at base-line achieved a CSS difference in the short term. Using the ODI for perceived low back disability from pain, we observed 43.9% at a level of SCS at follow-up for the entire sample, and 36.9% for those with CSS disability at baseline (≥10 points).

### Patients Reporting Symptomatic Reactions

One thousand eighty-nine of the 1,090 patients completed the SR survey at follow-up. Five hundred seven (46.5%) patients reported no SRs following UC care. Five hundred eighty-three (53.5%) patients believed they experienced at least one SR with a NRS rating of 0-10 following any of their UC adjustments. Three hundred thirty-eight (31.0%) patients reported SRs meeting the accepted definition (a new symptom not present at baseline or a worsening of a presenting complaint by >30% occurring ≤24 hours). Fifty-six (5.1%) patients reported at least one intense SR following UC care with a NRS ≥8.

### Symptomatic Reactions Reported by Patients

A total of 1,456 SRs with a NRS between 0-10 were reported by 583 patients. Forty-four SRs were rated "0" and 216 rated "1" using NRS for pain/discomfort intensity. Seven hundred eighty-seven (54%) of the 1,456 reported SRs began ≤24 hours following care with a NRS ≥1 and 637 (43.8%) met the definition of SR. Ninety-six (6.6%) of 1,456 reported SR that had a NRS ≥8. Eight hundred forty-two (57.8%) SRs were related to the nervous or circulatory systems with a mean intensity of 3.7 NRS. Five hundred fifty-four SRs of the 1,456 (38.1%) were musculoskeletal in nature with a mean intensity of 3.4 NRS, affected their daily activities "a little" with a duration of ≤24 hours consisting of primarily neck or lower back pain on average. Sixty SRs (4.1%) were psychological in nature with a mean intensity of 3.6 NRS. The 5 most frequent SRs meeting the accepted definition following UC chiropractic care with the percentage of occurrence were; Tiredness (10.4%), radiating pain (6.3%), neck pain (5.4%), dizziness (4.9%) and headache (4.2%) all primarily with mild intensity, short duration and little affect on daily living. In summary 338 (31.0%) patients reported 637 SRs meeting the accepted definition with no reports of serious SRs or serious AEs. Table 5 provides a summary of SRs following UC care.

**Table 5:** Frequency distributions and means (SDs) of symptomatic reactions (SRs), by type of reaction

SR Type Musculoskeletal	Patient Reported SR	**Intensity Mean (SD) 11-pt**.	SR by Definition	% of All SRs	Intense SR ≥ 8 NRS	%
Neck	394	3.4 (2.2)	79	5.4	22	2.0
Lumbar	129	3.4 (2.1)	21	1.4	7	0.6
Thoracic	13	3.8 (2.2)	3	0.2	2	0.2
Other*	18	4.6	11	0.8	2	0.2

*Clavicle, Knee, TMJ, Physical Activity Restriction, Foot, Spasm, Shoulder, Hip, Groin						

						

**SR Type Neurological Circulatory**	**Patient Reported SR**	**Intensity Mean (SD) 11-point**	**SR by Definition**	**% of All SRs**	**Intense SR **≥ **8 NRS**	**%**

Tiredness	210	3.6 (2.2)	151	10.4	13	1.2
Radiating	142	4.1 (2.4)	92	6.3	12	1.1
Headache	201	3.8 (2.3)	61	4.2	17	1.6
Dizziness	115	3.1 (2.2)	71	4.9	6	0.6
Arm/Leg Weakness	53	4.0 (2.3)	39	2.7	4	0.4
Tinnitis	48	3.2 (2.3)	25	1.7	3	0.3
Nausea	31	3.6 (2.3)	23	1.6	1	0.1
Blurred Vision	22	3.3 (1.7)	16	1.1	0	0
Numbness	4	4.5 (2.9)	2	0.1	1	0.1
Ear	4	4.0 (4.1)	2	0.1	1	0.1
Fainting	2	4.0 (5.7)	1	0.1	1	0.1
Other*	10	3.4	6	0.4	1	0.1

*Sinus, Abdominal, Heat, Eye, Shortness of Breath						

						

**SR Type Psychological**	**Patient Reported SR**	**Intensity Mean (SD) 11-point**	**SR by Definition**	**%**	**Intense SR **≥ **8 NRS**	**%**

Confusion	30	3.2 (2.1)	19	1.7	1	0.1
Depression	25	3.7 (2.9)	12	1.1	2	0.2
Mood	2	3.0 (2.8)	1	0.1	0	0.0
Sleep	2	6.0 (1.4)	1	0.1	0	0.0
Focus	1	7.0	1	0.1	0	0.0

Symptomatic Reactions (SR) reported and defined. Patients may report SRs that do not meet the accepted definition of SR, i.e. "A new symptom not present at baseline or a worsening of a presenting complaint by >30%." This table list reported SR with the mean intensity and SR by definition for presenting complaints of musculoskeletal, neurological/circulatory or psychological origins.

### Patient Satisfaction

Patients reported a very high degree of satisfaction with UC chiropractic care scoring a mean of 9.1/10 using an 11-point NRS. Patients that perceived they had any symptomatic reactions (SR) were 23% less likely to choose a satisfaction level of "Excellent", 13% less likely to choose "Very Good" or better and 6% less likely to choose the satisfaction level of "Good" or better (p ≤0.01). The data indicated a patient with a perceived SR was 19% more likely to choose a "Poor" satisfaction level (95% confidence interval 0.78 - 1.79). Table 6 lists estimated effects of SR on levels of patient satisfaction.

**Table 6:** Estimated effects (risk ratios [RRs] and 95% confidence intervals [CIs]) of SRs on levels of satisfaction* (n = 1089)

	Satisfaction = 10 (Excellent)	**Satisfaction **≥ **9** (Very Good)	**Satisfaction **≥ **8** (Good)	**Satisfaction **≥ **7** (Fair)	Satisfaction < 7 (Poor)
Type of SR	RR 95% CI	RR 95% CI	RR 95% CI	RR 95% CI	RR 95% CI
**Any SR**	0.77 0.70, 0.84	0.87 0.82, 0.93	0.94 0.90, 0.99	0.99 0.95, 1.02	1.19 0.78, 1.79
**Start **≤**24 hr & NRS > 1**	0.80 0.72, 0.88	0.90 0.83, 0.96	0.98 0.93, 1.03	1.01 0.97, 1.04	0.93 0.61, 1.41
**NRS **≥**8**	1.02 0.84, 1.25	0.97 0.83, 1.15	0.95 0.84, 1.07	0.99 0.91, 1.07	1.15 0.49, 2.73

## Discussion

This study provides the incidence of SRs related to UCT and the improvement in clinical outcomes following this form of spinal care. The preponderance of the evidence shows that major complications resulting from chiropractic care are very rare, although transient discomfort and other minor side effects of chiropractic care are common. Various prospective studies have shown about 30% to 60% of patients receiving SMT experience minor side effects shortly after treatment [1-12,38]. Local discomfort in the treatment area accounts for half to two-thirds of the reactions. Other effects such as radiating symptoms, headaches, tiredness/fatigue each account for about 10% of SRs. Side effects such as nausea, dizziness and other symptoms are rare and most studies show that each of these comprise of 5% or less of reported symptoms. The majority of reactions have been reported to begin within 24 hours of the treatment visit and to resolve in less than 24 hours.

Our current study compares favorably to most of these results. Thirty-one percent of the patients had SRs that were defined as the following:

• A worsening of presenting chief complaint of >30%

• A SR that has discomfort rating of >1

• A SR that began ≤24 hours from the patient's adjustment

This study compared the musculoskeletal SRs with the patients' presenting complaints. By using the clinical data of patients that presented with musculoskeletal symptoms (neck, headache, midback and/or lowback pain) (1035/1090 which is 95% of the patients) it was determined that only 12.3% of the patients had NRS that were >30% compared to their initial discomfort ratings (using the 11-point NRS for pain). While analyzing clinical data measurements this number may represent (1) SR, (2) the care failure rate or (3) a combination of the two. The study found the incidence of intense SR (≥8 on the NRS) was only 6.6% of the total number of SRs and occurred in 5.1% of patients. It should be noted the 83 chiropractors in the study were asked to estimate the number of upper cervical adjustments they had performed in their career. The total estimation for career adjustments came to 5,085,014. The doctors did not report any serious AE ever occurring in their practices (i.e., strokes or permanent injuries).

It should be noted that several of the side effects reported in our study have been found in previous studies with patients taking medication. Headaches, fatigue, dizziness, and nausea are among the most common drug-related adverse reactions [39], and these have been reported by people not taking any medication [40,41] The tenuous association between cervical SMT and cerebrovascular incidents (CVI) has been reviewed by various studies, and appears to have an estimated occurrence ranging from no causative association, to 1 in 300,000 to 500,000 [42,43], to 1.3 million [44], or 5.85 million cervical manipulations [45]. There appears to be a link between upper cervical rotation SMT and cerebrovascular incidents [46,47]; however, two cases have been reported in the literature involving strokes following non-force and neutral position cervical manipulative procedures [48,49]. Cassidy et al. [50] found that the risk of having a stroke was equal between patients consulting a chiropractor or general medical practitioner. This suggests that cervical SMT may not be a cause of cerebrovascular accidents, but associated with a stroke in progress. Our present study was not designed specifically to detect rare major complications, although doctors were responsible for reporting any and all AEs that occurred during the course of care.

The current study's outcome statistics are very similar to the results of a smaller case series (N = 66) studying neck pain outcomes following a low-force UCT [34]. The case series showed 34.8% of cases achieved a normal NDI (<5 or <10 for the 50 or 100 point scale, respectively) after 13.6 days, 5.7 office visits and 2.7 UC adjustments from a baseline of 3%, a 31.4% net gain. The current study shows 42.4% at a normal NDI from a baseline of 10.3% a 32% net gain. One prior study with a large number of patients (N = 529) studying AEs and outcomes following SMT demonstrated an 11.6% net gain score of NDI in the short term (4th visit) and 31.4% net gain in the long term (12 months/9.3 SMT treatments) [5]. 

The International Chiropractors Association published 'Best Practices and Practice Guidelines' in which they summarized multiple randomized controlled trials that used SMT or mobilization as the primary form of care and NRS or visual analog scale (VAS) as outcomes [51]. They summarize 66 RCTs for low back pain including a total of 4,661 patients, with 36 RCTs reporting NRS or VAS. Twenty-four RCTs involved chiropractors. The average number of office visits was 8.3 reporting a 42.6% improved NRS for pain on average. For neck, upper back and headache pain they summarize 54 RCTs (23 with chiropractors) including 2,069 patients reporting an average improvement of 46.5% with 7.7 office visits [5,8,52-55]. The current study differs in that it is not an RCT but a prospective, multicenter observational cohort study and cannot establish a causative association; however, it is similar in nature due to the large numbers of patients involved. A direct comparison cannot be made due to differing patient populations and other reasons.

It can be said that UCT fairs well when judged against the published guidelines and other studies in terms of patient safety and clinical efficacy. One of the chiropractic premises is that the correction or reduction of relative vertebral misalignment(s) in the upper cervical spine is clinically important. However, some believe the concepts are invalid, insignificant or implausible as it relates to outcomes, especially for spinal conditions like lower back pain because it is not adjacent to the upper cervical region. The data in the current study demonstrates a 57% average improvement for lower back pain following UCT but reports only 1.4% SRs in the lower back region. This data stimulates the question: "Is UCT a plausible type of care for lower back pain patients?" Two case series and one randomized clinical trial have demonstrated better outcomes for musculoskeletal symptoms and hypertension (without pain), respectively, for patients receiving reductions in C1 misalignment following UCT care relative to patients receiving smaller or no corrections in alignment [33,34,56]. Also, the present study reports fewer adjustments and a shorter follow-up period than previous chiropractic studies while demonstrating similar or better-improved outcome levels [52-55].

Two studies have demonstrated a statistically better clinical outcome in patients following Grostic/Orthospinology care [34,56]. This was achieved in the patients with a significant reduction in upper cervical misalignments as measured on their initial post adjustment radiographs. The patients with higher percent corrections on the first visit had a decreased need for follow-up visits and upper cervical adjustments. The subjects receiving higher upper cervical misalignment corrections also demonstrated higher improvement in various clinical outcomes compared to those having a lesser degree of correction. However, both of these studies were retrospective in design and did not have control groups.

Patient satisfaction in this study was very high with an overall rating of 9.1 on the 11-point NRS. Hertzman-Miller, et al. [57] found an average patient satisfaction rating of 30.6 (7.1)/36.1 (5.4) for medical and chiropractic care, respectively, for lower back pain patients on a scale of 10 (least satisfied) to 50 (most satisfied). Rubinstein et al. [5] reports a patient satisfaction rating of 7.8 (1.8) on an 11-point NRS for neck pain patients undergoing a trial of primarily diversified chiropractic care after 3 visits. Hurwitz et al. [58] found greater satisfaction increased the odds of remission from clinically meaningful pain and disability at 6 weeks but not at 6, 12 or 18 months. The current study revealed higher risk ratios for a satisfaction rating of poor for patients with any SR and intense SRs after an average of 17 days of care. Other studies and our data indicate that SRs and outcomes for pain and dysfunction are inversely associated in the short term while patient satisfaction and outcomes have a direct relationship.

This study has several strengths, including a large sample size, wide geographic representation, high participation and follow-up rates, and use of validated outcome measures; however, the observational (non-randomized) nature of the study precludes firm causal inferences of upper cervical chiropractic care with clinical outcomes, symptomatic reactions and patient satisfaction. Because the study lacks a control group, we could not estimate risks and rates of outcomes relative to other types of care or to a no-treatment condition. Also, our findings may not necessarily apply to the provision of other types of chiropractic care, care delivered in other settings, or in populations of patients with other conditions, comorbidities, or with different prognoses. Nevertheless, our study offers a valid description of the symptomatic reactions, clinical outcomes and patient satisfaction associated with UC chiropractic care in a cohort of over 1,000 patients.

## Conclusion

Chiropractic care using UCT may have a fairly common occurrence of mild intensity SRs short in duration (<24 hours), and rarely severe in intensity; however, outcome assessments were significantly improved with less than 3 weeks of care with a high level of patient satisfaction. Although our findings need to be confirmed in subsequent randomized studies for definitive risk-benefit assessment, the preliminary data shows that the benefits of UC chiropractic care may outweigh the potential risks.

## Competing interests

Authors KE and RPR are members of the board of the Society of Chiropractic Orthospinology, Inc. (SCO), which is a non-profit organization that teaches postgraduate courses to chiropractors and helps fund research studies. The SCO does not pay KE or RPR for their services but reimburses them for some of their expenses related to their work for the organization. Eric L. Hurwitz, D.C., Ph.D. (ELH) was hired as a consultant for this study and to conduct statistical analysis.

## Authors' contributions

All authors were involved in the design, conduct, and analysis of the study. KE obtained IRB approval and directed participant recruitment and data collection; RPR oversaw data management and quality control; and ELH conducted the primary statistical analysis. KE was the primary author of the first draft; RPR and ELH drafted sections of the manuscript and revised the manuscript through several drafts. All authors approved the final version.

## Pre-publication history

The pre-publication history for this paper can be accessed here:

http://www.biomedcentral.com/1471-2474/12/219/prepub
